# Brain-Scale Electroencephalography Patterns of Sleep and Wake in Schizophrenia

**DOI:** 10.1016/j.bpsgos.2024.100428

**Published:** 2025-01-15

**Authors:** Alena Damborská

**Affiliations:** Department of Psychiatry, University Hospital Brno and Faculty of Medicine, Masaryk University, Brno, Czech Republic; Brain and Mind Research Program, Central European Institute of Technology, Masaryk University, Brno, Czech Republic

Sleep-related pathology in schizophrenia has been reported since its early clinical description, when Emil Kraepelin noticed disrupted sleep in patients with dementia praecox, the early definition of schizophrenia. To date, most of the research using electroencephalography (EEG) recordings during sleep in schizophrenia has focused on changes in large-scale (across brain regions) neural oscillations, such as sleep spindles. These bursts of 11- to 15-Hz activity that occur during stage 2 non–rapid eye movement sleep are reduced in patients with schizophrenia (1).

SEE CORRESPONDING ARTICLE NO. 100371

In addition to these functional observations, a loss of gray matter was found in schizophrenia, which was proposed to represent the structural loss of synaptic connections among neurons as a result of abnormal pruning of dendrites during development (2). Eventually, with the emergence of network neuroscience in recent decades, schizophrenia has been conceptualized as a dysconnection syndrome (3). Given that spindle-related functional communication has only been identified among certain brain regions (4), the use of this frequency-based EEG phenomenon may be insufficient to investigate any schizophrenia-related whole-brain changes in functional connectivity. From this perspective, the study by Murphy *et al.* (5) just published in *Biological Psychiatry: Global Open Science* elegantly combines the large-scale and brain-scale approaches to investigate EEG abnormalities during sleep and wake in schizophrenia. Besides exploring the sleep spindles, the authors used microstate analysis employing the latter approach.

In the microstate technique, EEG data can be modeled as consisting of a small number of characteristic scalp potential topographies that persist for periods of around 100 ms and then transition to a different characteristic topography (6). These periods of quasi-stable topography are referred to as functional brain microstates, and their temporal dynamics are considered to reflect the process of global integration at the whole brain–scale level. Interestingly, the microstate approach is considered to be a promising tool to study both instantaneous and lagged manifestations of network dynamics in EEG (7), thus opening wide perspectives in functional connectivity research. Progress in brain topography using microstates in recent decades has raised hopes that noninvasive EEG can help increase our understanding of dysconnectivity in human brain diseases, including schizophrenia. In this regard, the findings by Murphy *et al.* (5) provide an important evidence of EEG abnormalities in schizophrenia.

Using high-density EEG data from 114 patients with schizophrenia, Murphy *et al.* (5) demonstrated behavioral state–dependent patterns of whole-brain network electrophysiological activity in this population. In the overlapping dataset, the authors of the current article have previously shown that patients with schizophrenia have decreased sleep spindles during sleep compared with healthy control participants (8). In the current article, Murphy *et al.* (5) therefore hypothesized that microstates C and E, whose topographies resemble the topography of sleep spindles, might be decreased in schizophrenia. Microstate E showed decreased presence in their sleep data. Unexpectedly, however, the results revealed increased microstate C presence in patients during non–rapid eye movement sleep. These findings support the utility of the microstate approach in schizophrenia research, providing distinct new information on electrophysiological abnormalities in schizophrenia beyond the existing frequency-based discoveries.

Interestingly, unlike previous reports on quiet resting-state microstates in patients with schizophrenia, Murphy *et al.* (5) found neither increased microstate C nor decreased microstate D during wake in this population compared with healthy control participants. Actually, the authors reported no difference in the presence of these microstates between patients and control participants and instead showed decreased microstate F duration during wake in patients. As Murphy *et al.* (5) indicated, and this interpretation does make sense, previous findings may not be replicated simply because of different methodological approaches being used. The authors of the current study progressively used a data-driven approach for the selection of number of microstates and did not rigidly stick to the canonical 4 microstates just for the sake of tradition in the microstate literature. Moreover, contrary to most of the previous microstate studies, Murphy *et al.* (5) used a single set of microstate topographies that was common for participant groups, behavioral states, and even frequency band filtering options.

The suggestion of Murphy *et al.* (5) that the approach of one-set-for-all-data topographies is appropriate is logical when searching for differences in microstate dynamics between conditions. Generally, when searching for the presence of an object in different environments, the same object in all environments can be detected based on some property that characterizes that object. In the case of a microstate, such a property is its topography, which when derived from all data enables the detection of the same microstate in all conditions. Temporal parameters of the detected microstate can subsequently be compared between conditions. On the other hand, using separate sets of topographies may reveal condition-specific microstates. In this respect, Murphy *et al.* (5) also argue, based on comparable explained variance in their sleep and wake data, that similar microstate topographies are produced in different behavioral states. Certain reservations about this conclusion may stem from the fact that a separate segmentation for each behavioral state was not performed in their study. In this regard, it would be interesting to see whether additional behavioral state–specific microstate topographies can be detected in their sleep or wake data. Nevertheless, as Murphy *et al.* (5) indicate, the 4 canonical wake-derived topographies are present in their current sleep data, supporting the hypothesis of a common set of brain activation patterns occurring during sleep and wake (9). Furthermore, as Murphy *et al.* (5) also admit, the interpretation of the microstate topography in relation to the cortical connectional architecture should be viewed with caution because this relationship has not been established yet (10).

For the same methodological reasons, there is also a need for some caution in interpreting the results of grossly unaltered microstate topographies in schizophrenia, as put forward by Murphy *et al.* (5). The direct evidence for such a statement awaits further investigation on the dataset, performing segmentation of microstate topographies separately for the case and control groups.

Finally, Murphy *et al.* (5) investigated the complexity of microstate transitions and found behavioral state–dependent case-control differences in complexity, which were greater during wake and smaller during non–rapid eye movement sleep in patients than in healthy control participants. Authors interpret this finding to mean that the transition structure is more complicated in patients than in control participants during wake and less complicated during sleep, suggesting distinct functional abnormalities in different thalamic nuclei in schizophrenia. Additional studies will need to be pursued to gain deeper insight into this interesting observation.

In conclusion, Murphy *et al.* (5) have a beautiful dataset with a large sample size and provide a sophisticated and cogent approach to exploring EEG changes in schizophrenia. The current study should inspire further microstate research that could open doors to new objective markers of psychiatric disorders, including but not restricted to schizophrenia.
